# Spin resolved electron density study of YTiO_3_ in its ferromagnetic phase: signature of orbital ordering

**DOI:** 10.1107/S2052252519009230

**Published:** 2019-08-02

**Authors:** Ariste Bolivard Voufack, Iurii Kibalin, Zeyin Yan, Nicolas Claiser, Saber Gueddida, Béatrice Gillon, Florence Porcher, Arsen Gukasov, Kunishisa Sugimoto, Claude Lecomte, Slimane Dahaoui, Jean-Michel Gillet, Mohamed Souhassou

**Affiliations:** a Université de Lorraine, Laboratoire CRM2, UMR CNRS 7036, Boulevard des aiguillettes BP70239, 54506 Vandoeure-les-Nancy, France; b CNRS, Laboratoire CRM2, UMR CNRS 7036, Boulevard des aiguillettes BP70239, 54506 Vandoeure-les-Nancy, France; c PNPI NRC, Kurcharov Institut, Orlova Rosha, Gatchina, Leningrad region 188300, Russian Federation; d CentraleSupelec, Grande Voie des Vignes, 92295 Chatenay-Malabry Cedex, France; e LLB, CEA-CNRS, UMR 12, CEA Saclay, 91191 Gif-sur-Yvette Cedex, France; f SPring-8, Japan Synchrtron Radiation Research Institut, 1-1-1 Kouto, Sayo, Hyogo 679-5198, Japan; g SPMS, UMR 8580, CentraleSupelec, Paris Saclay University, 91191 Gif-sur-Yvette, France

**Keywords:** perovskites, YTiO_3_, X-ray diffraction, polarized neutron diffraction, multipolar refinement, charge density, spin density, magnetic order, orbital ordering, computational modelling, inorganic materials, materials modelling, properties of solids

## Abstract

The present work reports on the charge and spin density modelling of YTiO_3_ in its ferromagnetic state (*T*_C_ = 27 K). Accurate polarized neutron diffraction and high-resolution X-ray diffraction (XRD) experiments were carried out on a single crystal at the ORPHÉE reactor (LLB) and SPRING8 synchrotron source. The experimental data are modelled by the spin resolved pseudo-atomic multipolar model (Deutsch *et al.*, 2012 ▸). The refinement strategy is discussed and the result of this electron density modelling is compared with that from XRD measured at 100 K and with density functional theory calculations. The results show that the spin and charge densities around the Ti atom have lobes directed away from the O atoms, confirming the filling of the *t*_2g_ orbitals of the Ti atom. The *d*_*xy*_ orbital is less populated than *d*_*xz*_ and *d*_*yz*_, which is a sign of a partial lift of degeneracy of the *t*_2g_ orbitals. This study confirms the orbital ordering at low temperature (20 K), which is already present in the paramagnetic state above the ferromagnetic transition (100 K).

## Abstract 2

The first joint refinement against X-ray and polarized neutron diffraction data performed to simultaneously model the charge and spin density of the perovskite YTiO_3_ in its ferromagnetic state is presented. The resulting model confirms the orbital ordering at low temperature.

## 1. Introduction   

There is increasing interest in materials having strong electron correlation. Many of these compounds are transition metal oxides (TMOs) with a perovskite structure where the transition metal ion is octahedrally coordinated by six O atoms. Though many of these TMOs are Mott insulators (MIs) with antiferromagnetic order, YTiO_3_ is one of the rare MIs with a ferromagnetic ground state. The titanate family *A*TiO_3_ exhibits different magnetic properties depending on the *A*-type cation. The two classical Mott–Hubbard insulators LaTiO_3_ and YTiO_3_ are formally isoelectronic with a 3*d* ^1^ electron configuration of Ti. YTiO_3_ orders ferromagnetically below the Curie temperature *T*_C_ ≃ 27 K, whereas LaTiO_3_ orders antiferromagnetically below the Néel temperature *T*_N_ = 150 K (Akimitsu *et al.*, 2001 ▸). In these systems, the unpaired electron is mostly localized on the Ti ion which is responsible for the magnetic properties. A change from ferromagnetism to antiferromagnetism can be continuously tuned by varying the lanthanum concentration (*x*) in the Y_1−*x*_La_*x*_TiO_3_ alloys or by changing the *A* cation in *A*TiO_3_ (Goral & Greedan, 1982 ▸; Knafo *et al.*, 2009 ▸).

**Figure 1:**
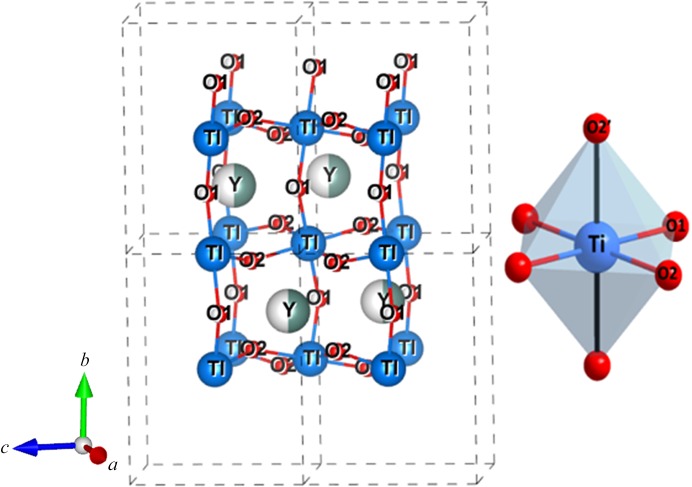
(Left) Crystal structure of YTiO_3_: O atoms in red and Ti in blue. (Right) Ti octahedron and local axes.

Electronically, in YTiO_3_ the Ti^3+^ ion has a formal 3*d* ^1^ electronic configuration; its fivefold degeneracy is broken due to the local crystal field effects produced by the surrounding octahedron of oxygens (see Fig. 1 ▸). It then results in two distinct groups of *d* electronic states. The first grouping is referred to as the *t*_2g_ electrons, the associated orbitals (d_*xz*_, d_*yz*_ and d_*xy*_) are directed away from the neighbouring O atoms. Due to this orientation, there is a minimal overlap with the valence electrons on the neighbouring oxygens and, as a consequence, these states tend to be lower in energy. The second grouping is the *e*_g_ electrons, associated with the 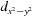 and 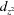 orbitals pointing towards the neighbouring oxygens; these states tend to be higher in energy and participate in covalency. Like many other perovskites, YTiO_3_ presents a GdFeO_3_-type distortion (Geller, 1956 ▸; Goodenough, 1963 ▸) that is driven by ion-size mismatch and induces rotations of the TiO_6_ octahedra. The distorted structure is caused by lowering the symmetry of the TiO_6_ octahedron away from the perfect cubic perovskite (like BaTiO_3_ at high temperature) to an orthorhombic structure (*Pnma*). This distortion is more pronounced in YTiO_3_ than in LaTiO_3_, favoured by smaller *A* ^3+^ ions such as Y (*r*_ionic_ = 1.04 Å) compared with La (*r*_ionic_ = 1.17 Å) (Knafo *et al.*, 2009 ▸; Pavarini *et al.*, 2005 ▸; Mochizuki & Imada, 2004 ▸; Leoni *et al.*, 2006 ▸). In YTiO_3_, an additional elongation (about 3%) of the TiO_6_ octahedron is observed compared with LaTiO_3_. This distortion has been ascribed to staggered ordering of the Ti *t*_2g_ orbitals (Akimitsu *et al.*, 2001 ▸; Iga *et al.*, 2004 ▸; Komarek *et al.*, 2007 ▸; Knafo *et al.*, 2009 ▸). The switch from antiferromagnetism to ferromagnetism in *A*TiO_3_ perovskites is probably controlled by the extreme sensitivity of the magnetic superexchange interactions to the distortions of the lattice (Knafo *et al.*, 2009 ▸; Pavarini *et al.*, 2005 ▸; Mochizuki & Imada, 2004 ▸; Solovyev, 2006 ▸). However, the mechanism driving this transition is still a matter of considerable debate (Pavarini *et al.*, 2005 ▸; Knafo *et al.*, 2009 ▸). In the last two decades, YTiO_3_ has been the subject of many studies using a variety of experimental methods and theoretical models (Suzuki *et al.*, 2007 ▸; Knafo *et al.*, 2009 ▸; Ulrich *et al.*, 2009 ▸; Ichikawa *et al.*, 2000 ▸; Akimitsu *et al.*, 2001 ▸; Itoh *et al.*, 1999 ▸; Nakao *et al.*, 2002 ▸; Varignon *et al.*, 2017 ▸).

Recently we have confirmed Akimitsu’s (Akimitsu *et al.*, 2001 ▸) and Itoh’s (Itoh *et al.*, 1999 ▸) results using the joint refinement of polarized neutron diffraction (PND) and X-ray magnetic diffraction (XMD) data, showing that the Ti^3+^ 3*d* ^1^ wave­function can be described by a linear combination of *d*_*xz*_ and *d*_*yz*_ orbitals (Kibalin *et al.*, 2017 ▸). The reconstructed spin density in momentum space, using either theoretical calculations or the experimental Compton profiles, is in very good agreement with the description in direct space (Yan *et al.*, 2017 ▸). The present paper is devoted to the precise determination of the electronic state at low temperature which is absolutely necessary to understand the electronic properties of YTiO_3_ as the Ti^3+^ unpaired electron plays a crucial role in the control of its magnetic properties. The only attempt of charge density analysis on YTiO_3_ has been made by Hester at 127 K (Hester *et al.*, 1997 ▸) using W *K*α radiation (λ = 0.21069 Å) to reduce absorption and extinction effects. No multipolar analysis has been performed: only experimental deformation electron density maps revealed large charge depletions along the Ti—O bonds. In the present study, the electron density distributions of charge ρ(*r*) and of spin *s*(*r*) have been determined using high-resolution XRD and PND data simultaneously. Combining these two techniques in the refinement of a unique model provides the spin resolved electron density using the spin–split extension of the Hansen–Coppens model (Hansen & Coppens, 1978 ▸; Deutsch *et al.*, 2012 ▸, 2014 ▸).

The difficulties encountered to model the electron density are common to most pure inorganic crystals containing heavy elements: very low scattering power of the diffuse Y and Ti ion valence electrons compared with the core ones (ratio of 1/18 for Ti^3+^), important absorption and extinction effects. A parameter to evaluate the difficulty to experimentally probe charge density of such a heavy element is the suitability index (Stevens & Coppens, 1976 ▸). For a crystal material such as YTiO_3_, this index is very low (∼0.045) compared with coordination complexes (0.4–0.6) or organic compounds (3–5). This is an *a priori* indication on the intrinsic difficulty of modeling its charge density.

## 2. Experimental   

The X-ray diffraction experiment was carried out using the SPring-8 synchrotron radiation source (beamline BL02B2) on a single crystal (0.02 × 0.10 × 0.11 mm). A short wavelength of 0.3506 Å was used to reduce absorption and extinction effects. The data were collected at 20 K, which is 7 K below the ferromagnetic phase transition temperature (*T*_C_ = 27 K). The diffractometer is equipped with a cylindrical image plate which considerably enhances the signal-to-noise ratio. Data collection consisted of four scans at positions χ = 0, 15, 30 and 45° using a rotation angle of 11° (1° overlap between images) and 13 min exposure time. Due to overflow, 16 images were re-measured with 3 min exposure time. The indexing, intensity integration and the absorption correction were carried out using the in-house programme *RAPID AUTO* (Rigaku, 2009 ▸). A total of 100 406 reflections were collected, out of which, 96 986 reflections were averaged with *SORTAV* (Blessing, 1987 ▸) leading to 4584 unique reflections with an average redundancy of 21 and an internal agreement *R*_int_ = 3.66% up to sin θ_max_/λ = 1.67 Å^−1^. The agreement factor *R*_int_ increases smoothly with increasing resolution (Table S1 of the supporting information) to reach about 8% in the last shell (1.4–1.67 Å^−1^), attesting to the very good quality of the data. Such high-quality ultra-high resolution data allows a thorough modelization of the atomic displacement parameters (ADPs).

Polarized neutron diffraction measurements were carried out at the thermal polarized neutron lifting counter diffractometer 6 T2 (LLB-ORPHÉE, Saclay) at low temperature (5 K) using a 5 T external magnetic field to fully magnetize the sample. A total of 291 flipping ratios were measured and corrected from extinction effect to a maximal resolution of 0.5 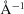. For more details, see the work of Kibalin *et al.* (2017 ▸). Table 1 ▸ summarizes the experimental and crystallographic data.

## 3. Thermal displacement and structural analysis   

### 3.1. Anharmonicity   

**Figure 2:**
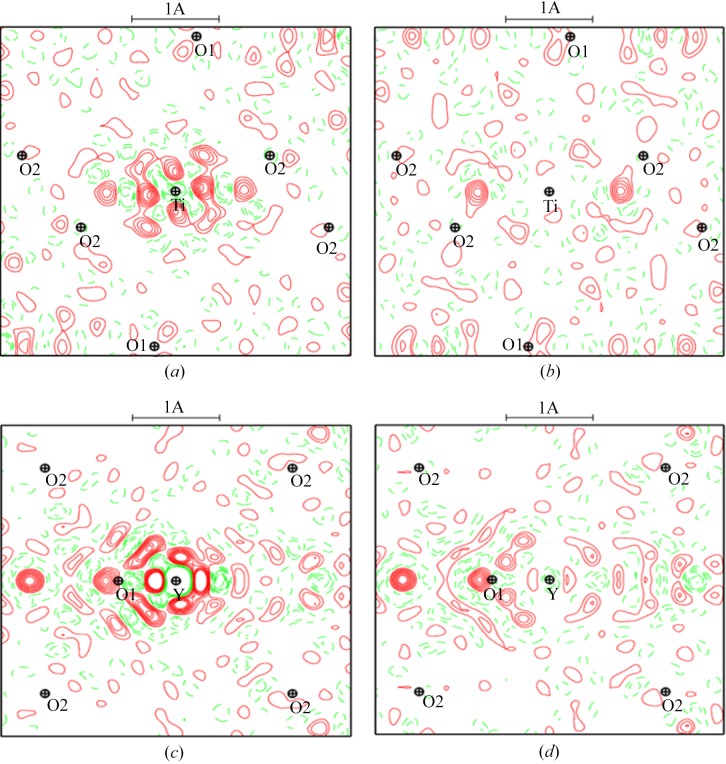
Residual density at high resolution (*N*_ref_ = 2549, sin (θ)/λ >1.25 Å^−1^) in the (001) plane containing Y and Ti atoms: (*a*) and (*c*) harmonic, and (*b*) and (*d*) anharmonic models. Contour: 0.2 e Å^−3^.

A first structural refinement with all data using harmonic ADP showed large residual electron densities around heavy atoms as depicted in Fig. 2 ▸ (upper). These residues are still very large at high θ angles (where only core electrons scatter) and are structured as alternating positive and negative densities in the crystallographic (001) plane (see also Fig. S1 of the supporting information for other planes), suggesting anharmonic vibration of the heavy atoms or core deformation. Therefore, the refinement of anharmonic ADPs of Ti and Y atoms, modelled by Gram–Charlier coefficients up the 6th order, was carried out at very high resolution (1.2 < sin θ/λ < 1.668 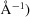. The residues around the concerned atoms are reduced drastically as shown in the bottom part of Fig. 2 ▸. Residual maps around O atoms are clean and do not show any anharmonicity. The statistical agreement factors for all 4584 data significantly dropped from *R*(*F*) = 2.48%, *R*_w_ = 2.58% and GooF = 2.83 for the harmonic model to *R*(*F*) = 1.22%, *R*_w_ = 1.39% and GooF = 1.53. Significant parameters (30/86 > 3σ) are summarized in Table S2. Anharmonicity in YTiO_3_ has not been described; it was not observed at 100 K for which the resolution was lower sin θ_max_/λ = 1.28 Å^−1^ (Voufack, 2018 ▸) nor at 127 K (Hester *et al.*, 1997 ▸), but was observed in other perovskites when the experiment temperature is close to the transition temperature such as in PbTiO_3_ (Kiat *et al.*, 2000 ▸), KNiF_3_ (Ivanov *et al.*, 1999 ▸), KMnF_3_ (Ivanov *et al.*, 2004 ▸) and CsPbCl_3_ (Hutton & Nelmes, 1981 ▸). Anharmonicity in SrTiO_3_ has been controversial for some time. Jauch used high-resolution γ-ray diffraction to show that a harmonic model was good enough to fit their data (Jauch & Reehuis, 2005 ▸). However, a recent study showed an increase of significant anharmonic displacements for all atoms when the temperature approaches the transition temperature (Yamanaka *et al.*, 2017 ▸). One main difference with cited studies is about the ADP of the O atoms. Hutton & Nelmes (1981 ▸) claimed, using neutron diffraction, that anharmonicity affects more anions than cations. In YTiO_3_, no significant anharmonic ADPs were detected for O atoms.

### 3.2. Structural analysis   

Fig. 1 ▸ shows the structure of YTiO_3_. The Ti^3+^ ion sits on the centre of a centrosymmetric distorted oxygen octahedron. The distances (Table 2 ▸) between Ti and O atoms are *d*_(Ti—O1)_ = 2.0164 (8), *d*_(Ti—O2)_ = 2.0194 (9) and *d*_(Ti—O2′)_ = 2.0784 (7) Å (apical axis). The angles O1—Ti—O2 and O2—Ti—O2′ remain close to 90° [89.51 (2) and 89.37 (1)°, respectively] whereas O1—Ti—O2′ is 86.62 (2), about 3.4° away from 90°. The joint angles linking the Ti octahedra are 140.10 (3)° for Ti—O1—Ti and 143.73 (2)° for Ti—O2—Ti, showing the distortions and different orientations of the Ti octahedra. The Ti—O distances at 20 K are slightly shorter than those at 100 K (0.003Å ≃ 3σ) (Voufack, 2018 ▸) and the joint angles do not change. All these small changes between 100 and 20 K structures are due to the thermal contraction. The Y^3+^ ion sits on a mirror plane and is coordinated by eight O atoms forming a distorted square antiprism, with distances ranging from 2.234 (1) to 2.6826 (5) Å (Table 2 ▸). The Y atom has four short contacts, two with O1 atoms [2.234 (1) and 2.310 (1) Å] and two with O2 atoms [2.2778 (7) Å]. The other four contacts with O2 atoms are longer [2.501 (1) and 2.677 (1) Å]. Coordination angles around the Y atom range from 79.60 (2) to 153.98 (1)°. The variations of distances with respect to 100 K are negligible. The O1 atom also lies on the mirror plane and interacts with two Ti and two Y atoms, forming a distorted irregular tetrahedron. O2 is linked to two Ti and three Y atoms with three short distances and two longer ones (Table 2 ▸). After passing the phase transition, the geometrical parameters do not significantly change when the temperature decreases from 100 to 20 K.

## 4. Spin resolved electron density modelling   

### 4.1. Methodology   

The experimental spin resolved electron density distribution was modelled using the program *MOLLYNX* (Deutsch *et al.*, 2012 ▸), a modified version of the Hansen–Coppens formalism (Hansen & Coppens, 1978 ▸; Deutsch *et al.*, 2014 ▸) where the pseudo-atomic spin resolved electron density is expanded on real spherical harmonic functions (*y*_*lm*±_) for magnetic atoms. In this formalism the charge density is expressed as: 
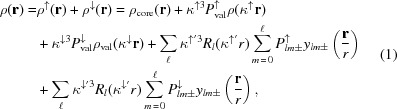
and the spin density is expresssed by:
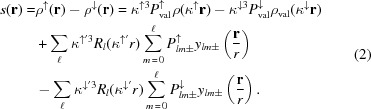
ρ_core_ and ρ_val_ are core and valence contributions to the charge density, respectively; *P*_val_ and *P*_*lm*±_ are valence and multipolar parameters for electrons, respectively, with spin up (↑) and spin down (↓). The radial function 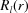 is a Slater-type function (see Table S3 for initial parameters). The radial functions ρ_val_(*r*) and *R*_*l*_(*r*) are modulated by κ and κ′ (contraction/expansion). For atoms carrying magnetic moments the density parameters are split into up (↑) and down (↓) according to equation (1). For atoms without magnetic moments the standard Hansen and Coppens model is used. The advantage of this model is the simultaneous determination of spin resolved density by joint refinement of XRD and PND data. In YTiO_3_, the unpaired electron is mainly located on the Ti atom, so all Ti population parameters (*P*_val_ and *P*_*lm*±_) were split, whereas only monopoles (*P*_val_) were split for other atoms to account for possible spin transfer or polarization. The local axis of the Ti atom involves *x* along the Ti—O1 direction, *y* along the Ti—O2 short directions and *z* close to the Ti—O2′ longest bond (within 3.4°) (Fig. 1 ▸).

### 4.2. X-ray refinement   

First, a multipolar refinement was performed against X-ray data only. In the independent atom model (IAM), the neutral valence shells were assigned 5*s* ^2^4*d* ^1^ for Y, 4*s* ^2^3*d* ^2^ for Ti and 2*s* ^2^2*p* ^4^ for O atoms. The radial scattering was calculated using the neutral atom wavefunctions of Clementi & Roetti (1974 ▸) for O, and Thakkar & Toshikatsu (2003 ▸) for Y and Ti. The isotropic extinction parameter was refined using the Becker and Coppens formalism (Becker & Coppens, 1974 ▸). The (121) reflection is the most affected (*y* = 0.74 with Icorr = *yI*_meas_).

The distributions of 4*s* (Ti) and 5*s* (Y) electrons have very diffuse character. Fig. S2 shows the IAM valence scattering factors of Y and Ti independent atoms; 4*s*, 5*s* and 4*d* valence electrons contribute only at very low resolution (sin θ/λ < 0.2 Å^−1^), which makes them very hard to model experimentally. For Y only nine reflections contain the contribution of the valence scattering. In addition, these reflections are usually affected by extinction. Some authors either distribute these outer electrons on the ligand or fix them (Jauch & Reehuis, 2005 ▸). In this study, the valence scattering factor for Ti and Y atoms were chosen as a weighted linear combination of *s* and *d* electrons: 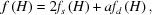
with *a* = 1 or 2 for Y and Ti, respectively. The X-ray-only multipolar refinement was first conducted using reflections with sin θ/λ < 1.2 (refined parameters are first *P*_val_ and κ, then *P*_*lm*±_ up to hexadecapoles for all atoms and finally the radial contraction/expansion κ′). This is followed by recycling between high-order, sin θ/λ > 1.2 (*xyz*, *u*_*ij*_, *c*_*ijklmn*_), and lower-order, sin θ/λ < 1.2 (*P*_val_, *P*_*lm*±_, κ, κ′), refinements. At the end, all parameters were refined using all data (4584 reflections). The statistical agreement is excellent [*R*(*F*) = 0.9%, *R*_w_(*F*) = 1%, GooF = 1.28, as calculated from the SORTAV estimated variances for 4584 reflections]. This is the limit of the multipolar model for which all the parameters are allowed to vary without any constraints. We are currently developing an atomic orbital model which constrains the refinement to the wavefunction of valence electrons (Kibalin *et al.*, 2019, to be published).

### 4.3. Joint refinement of XRD and PND   

**Figure 3:**
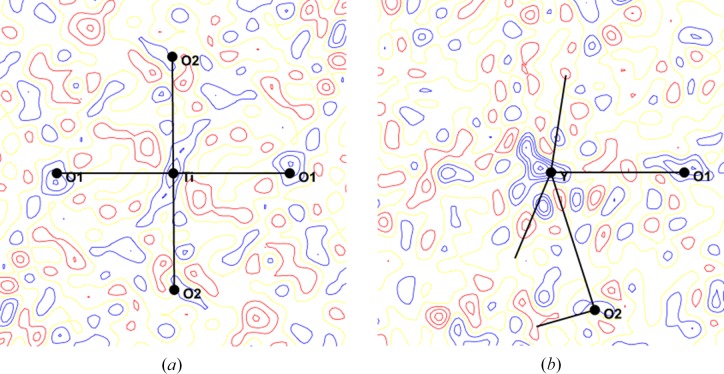
Residual density around (*a*) Ti and (*b*) Y atoms after the joint refinement. Contour: 0.1 e Å^−3^ sin (θ)/λ < 1.2 Å^−1^.

The multipolar model using X-ray data was only an initial guess for the joint refinement procedure combining XRD and PND data. A logarithmic weighting scheme (Deutsch *et al.*, 2012 ▸) was used to enhance the contribution of the 291 PND reflections compared with the 4584 XRD reflections. Multipolar parameters were constrained using 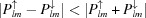, which insures that for any pole the density of unpaired electrons is less than that of the total electron density. For all atoms the valence and magnetic scattering factors were calculated using the neutral atom wavefunctions. The refined parameters are the monopole 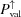 and 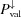 for all atoms and (*l*_max_ = 4) for the Ti atom. The splitting of κ and κ′ was carried out but did not improve the refinement. The final statistical agreement factors are summarized in Table 3 ▸. The X-ray residual charge density maps are calculated in different sections as shown in Fig. 3 ▸. The residues are reduced, with the maximum outside the mirror plane at about 0.2 e Å^−3^ (about 2σ), whereas in the mirror plane, the residues are slightly higher, with the maximum at about 0.4 e Å^−3^ (3σ) around the Y atom. These residues are not located on regions of contact between atoms. In the vicinity of Ti the maps show randomly distributed residues. The X-ray statistical agreement factors are excellent [*R*(*F*) = 1.11% and *R*_w_(*F*) = 1.36%, GooF = 1.34 for 4244 reflections; 0.79 and 1.0% for 1000 reflections with sin θ/λ < 1 Å^−1^ and *I* > 3σ(*I*)], attesting to the high quality of the data and model. The statistical agreement factors for PND are very good: *R*_w_(|1 − *R*|) = 11.6% and GooF = 9.7; all statistical indices are slightly larger than values obtained when the refinement is carried out on PND or X-ray data only (Kibalin *et al.*, 2017 ▸), which is to be expected as the model must be in agreement with both sets of data. The atomic fractional coordinates and anisotropic displacement parameters at the end of the multipolar joint refinement are given in Table S4.

## 5. Results and discussion   

### 5.1. Results   

This is the first successful attempt to map and model spin resolved electron density in a small unit cell pure mineral crystal.

The *P*_val_-κ derived charges (*Q* = *N*_val_ − *P*_val_) are usually less pronounced than formal ones. Refined valence and spin populations are summarized in Table 4 ▸. The Y atom has a valence population of *P*_val_ = 1.54 (7) leading to a net charge of +1.46 (7) compared with a formal +3 net charge. The Ti atom has a net charge 0.59 (6) instead of +3 formally. The O1 and O2 atoms have net charges of −0.66 (3) and −0.70 (2), respectively, similar to the values obtained in SrTiO_3_, *P*_val_(O) = 6.59 (Jauch & Reehuis, 2005 ▸) and rutile TiO_2_, *P*_val_(O) = 6.69 (Jiang *et al.*, 2003 ▸). The observed monopole population of the O atoms is then very similar to the cited literature between 6.5 and 6.75 e despite the different formal Ti oxidation states.

Charges are not uniquely defined and depend on the partitioning schemes – another way to estimate them is to integrate the total density over the atomic basins (Bader, 1990 ▸). The net atomic charges obtained using *Newprop* (Souhassou & Blessing, 1999 ▸) are summarized in Table 4 ▸, their values, +1.8, +1.5 and −1.0 for Y, Ti and O atoms, respectively, are slightly larger than the *P*_val_ ones. According to this estimation, YTiO_3_ is not a fully ionic system. The estimated atomic radii for Ti and Y atoms calculated as *R* = [(3/4π)*V*]^1/3^, where *V* is the volume of the atomic basin, (Table 4 ▸) are intermediate between ionic (Shannon & Prewitt, 1969 ▸) and covalent (Pyykkö & Atsumi, 2009 ▸) radii (see Table 4 ▸).

**Figure 4:**
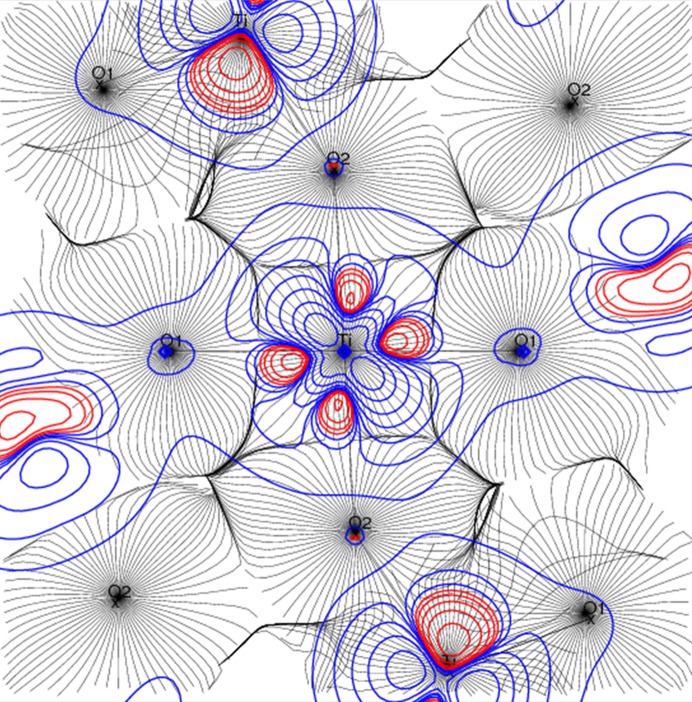
Electron density gradient map (black lines) defining the Ti and O atomic basins superimposed onto the spin density (positive in blue and negative in red using logarithmic contours), highlighting the spin density expansion towards the oxygen atomic basins.

The magnetic moment as deduced from the *P*_val_ estimation is mainly carried by the Ti atoms [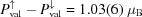]. Other atoms have negligible magnetic moments (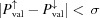). However, if the integration of the spin density is made on the atomic total density basins, all atoms carry a magnetic moment. Most magnetization is on the Ti atom (0.62 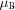), whereas the two O atoms have similar magnetic moments (0.1 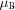) and the Y atom has a smaller value (0.07 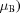. This unpaired electron partitioning using Bader atomic basins reflects the difference between the titanium refined valence population (+0.6 e) and AIM charges (+1.5 e) and hence their corresponding estimated volumes. Using AIM volumes to integrate spin is then counterintuitive in comparison with spin density maps as the unpaired *xz* and *yz* *d* electron density expands more than 1 Å away from the Ti nucleus. Fig. 4 ▸ gives the spin density in the O1—Ti—O2 plane superimposed to the Ti and O electron density gradient lines which define the atomic basins. The titanium 3*d* spin density lies mostly in the Ti atomic basin but expands also on the O1 and O2 atomic basins; this explains the non-zero spin density integrated over the O atomic basins. The oxygen AIM spin density is partially in line with our previous paper (Kibalin *et al.*, 2017 ▸) which showed that the magnetic pathway involves the O1 atom but not O2.

**Figure 5:**
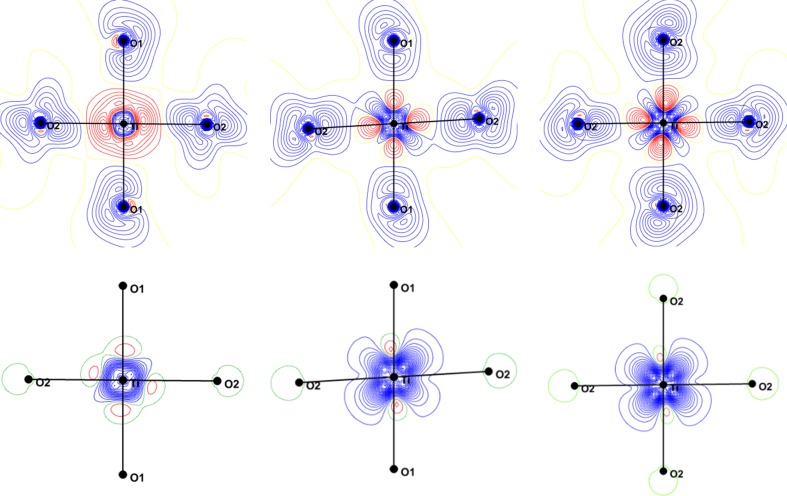
Static deformation densities (top) and spin (bottom) densities in the *xy* (left), *xz* (middle) and *yz* (right) planes containing the Ti atom. Contour: 0.05 e Å^−3^ for charge and 0.03 e Å^−3^ for spin densities.

**Figure 6:**
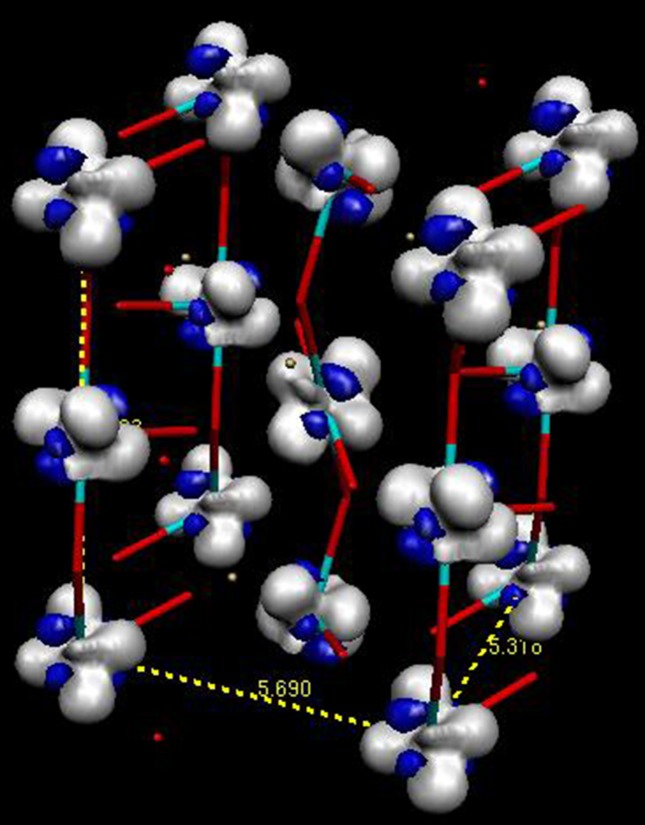
Isosurface spin density in the unit cell. Contour: 0.03 e Å^−3^.

**Figure 7:**
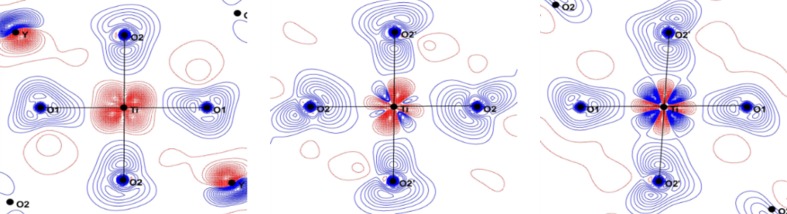
Static deformation density (at 100 K) in the *xy*, *xz* and *yz* planes (left to right). Contour: 0.05 e Å^−3^.

The static charge deformation density around the Ti atom is shown in Fig. 5 ▸ (upper panels). The accumulation of the deformation density is mainly located in the O—Ti—O diagonal directions; large positive lobes, in the *xz* and *yz* planes, directed at almost 45° from the Ti—O directions accompanied by large depletions in the direction of O atoms. The deformation charge density in the *xy* plane is more isotropic. The deformation density in the *xz* and *yz* planes is the signature of the population of *xz* and *yz* *d*-type orbitals. In fact, the maximum of the deformation density is out of these planes (Fig. 6 ▸), resulting from the combination of *xz*- and *yz*-type orbitals, which is called ordering in most papers related to the electronic structure of YTiO_3_. The Laplacian maps (Fig. S3) show similar features with electron concentration close to the Ti atom directed away from the O atom directions in the *xz* and *yz* planes, but the distribution is isotropic in the *xy* plane. The oxygen lone pairs are directed towards the Ti atoms; the maximum deformation density is obtained along the longest Ti—O2′ distance (2.078 Å) and the minimum for O1 that has the shortest distance to Ti (2.017 Å). The oxygen lone pair distribution is similar to the density observed in Ti^3+^ of Ti_2_O_3_ (Vincent *et al.*, 1980 ▸) and does not reveal as much covalency as in Ti^4+^ oxides. The deformation density around Y atom is very difficult to analyse (as few reflections can be used to model it, see above), it has a large quadrupole form; the positive and negative parts are not directed toward O atoms. However, the deformation density maps of the Y⋯O interactions (Fig. 7 ▸) show the polarization of the oxygen lone pairs toward Y. The positive deformation density lobe is pointing towards the Y atom and the negative part towards the voids.

The topological analysis of the total electron density (Table 5 ▸) shows that both short Ti—O contacts have the same topological properties that are different from the longest ones, their density at the bond critical points (in the middle of Ti—O bonds) is 0.6 e Å^−3^, which is 0.1 e Å^−3^ larger than the longest contact. The density at the critical points around Y atoms presents higher values than Ti for the short contacts (ρ_CP_ = 0.65 e Å^−3^); the density is high also for the longest interactions (ρ_CP_ = 0.24 e Å^−3^). These high densities at the Y and Ti critical points combined to the observed AIM charge reveal the partial covalent character of the Ti—O and Y—O contacts.

**Figure 8:**
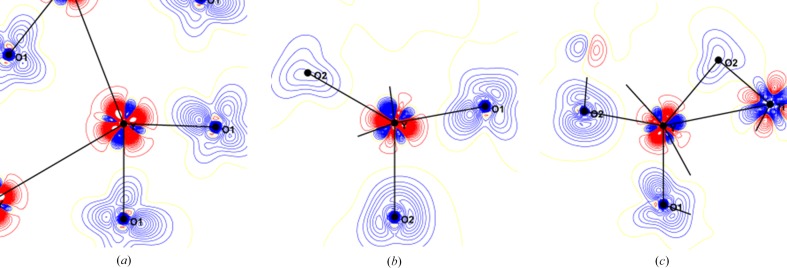
Static deformation density around the Y atom in the (*a*) mirror plane passing through O1,Y O1′, (*b*) the plane of Y, O1 and O2 short contacts, and (*c*) the plane of O1, Y and TI. Contour: 0.05 e Å^−3^.

The charge density of YTiO_3_ was also determined at 100 K (sin θ_max_/λ = 1.28 Å^−1^) using silver radiation (Voufack, 2018 ▸), resulting static deformation densities are shown in Fig. 8 ▸ in the *xy*, *xz* and *yz* planes. At 100 K, the positive deformation density around Ti is also mainly due to the *t*_2g_ *xz* and *yz* orbitals, showing already the partial degeneracy of *t*_2g_ orbitals and the corresponding orbital ordering. Therefore, this orbital ordering does not signify ferromagnetic properties which is opposite to what is often proposed.

The static spin density in the same planes is given in the lower panels of Fig. 5 ▸; it shows that the large redistribution of the spin density is in the *yz* and *xz* planes. In the *xy* plane, there is some spin density which has an almost spherical shape with a small elongation in the *d*_*xy*_ bis­ecting direction. In fact, the maxima of the spin density are not in these principal planes but are above them as shown in Fig. 6 ▸. This observation confirms that the unpaired electron occupies an orbital which is a linear combination of the *d*_*yz*_ and *d*_*xz*_ orbitals. This is consistent with our previous results obtained using PND only (Kibalin *et al.*, 2017 ▸), theoretical calculations and magnetic Compton measurements (Yan *et al.*, 2017 ▸), and the X-ray magnetic diffraction of Itoh (Itoh *et al.*, 1999 ▸), in accordance with the distortion of Ti octahedron and crystal field effects (Varignon *et al.*, 2017 ▸; Okatov *et al.*, 2005 ▸).

**Figure 9:**
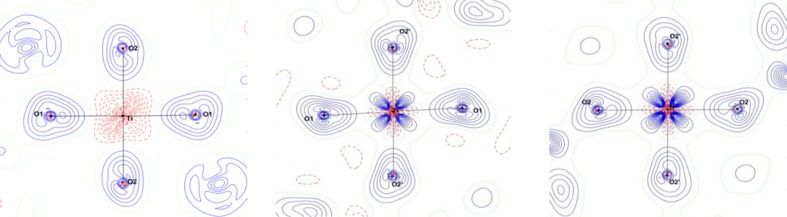
DFT charge deformation densities in *xy*, *xz* and *yz* planes (left to right). Contour: 0.05 e Å^−3^.

Theoretical calculations on YTiO_3_ were carried out using the *ab initio Crystal14* software for periodic systems at the DFT-PBE0-1/3 (Yan *et al.*, 2017 ▸). The resulting charge deformation density and spin density maps are shown in Figs. 9 ▸ and 10 ▸, respectively. These maps compare very well with the experimental ones. In the *xy* plane, the density is mainly spherical around Ti, in the *xz* and *yz* planes the lobes of the density are oriented in the bis­ecting direction of the Ti—O bonds. The lone pairs of O atoms are again facing the metal ions.

### 5.2. Discussion   

Jauch (Jauch & Reehuis, 2005 ▸), using γ-ray diffraction on SrTiO_3_, showed that the deformation density around the Ti^4+^ atom has maxima directed towards the O atoms, very similar to the results on TiO_2_ (Jiang *et al.*, 2003 ▸) and SrTiO_3_ (Friis *et al.*, 2004 ▸) (by combining electron diffraction and X-ray diffraction). Friis and Jiang stated that there is an indication that the two *e*_g_ orbitals hybridize with the O 2*sp* orbitals to form strong Ti—O σ bonds. The three *t*_2g_ orbitals hybridize with O 2*sp* to form weak Ti—O π bonds. They showed that band structure calculations agree well with the experimental values on the Ti—O polar covalent bonding. In these two compounds the average Ti—O distance is about 1.956 Å, much shorter than in YTiO_3_ where the minimum is 2.017 Å. In YTiO_3_, where the titanium ion is formally 3+, the deformation density accumulation is not directed towards the O atoms but in bis­ecting directions, which corresponds to the filling of two out of three *t*_2g_ orbitals. The low accumulation of the density towards O atoms is a sign for a lower covalency with low occupation of the *e*_g_ orbitals, but hybridization of unoccupied Ti *e*_g_ with O 2*p* orbitals still contributes to the Ti—O σ bond.

The charge density analysis around the Ti atom reveals charge depletion along the Ti—O bonds and accumulation in bis­ecting directions favouring the localization of electrons in the *d_xz_* and *d_yz_* sub-shells of 3*d* orbitals. The estimated *d* orbital populations from the titanium multipolar parameters, neglecting covalent effects (Holladay *et al.*, 1983 ▸), show that the orbitals *d*_*xz*_ and *d*_*yz*_ are the most populated (25 and 27%) and the remaining orbitals are almost even and less populated (16%, Table 6 ▸). The non-zero population of the *e*_g_ orbitals is due to the fact that 4*s* and 3*d* could not be refined separately yielding some *s* spherical contribution to all orbitals. If we subtract this *s* contribution, then the percentage occupancy of *d*_*xz*_ and *d*_*yz*_ becomes 67% and the other three orbitals are populated by only 10% each in accordance with an iono covalent Ti—O bond: the non-vanishing *e*_g_ population is the result of hybridization of the empty *e*_g_ of Ti with the oxygen 2*p* orbitals.

**Figure 11:**
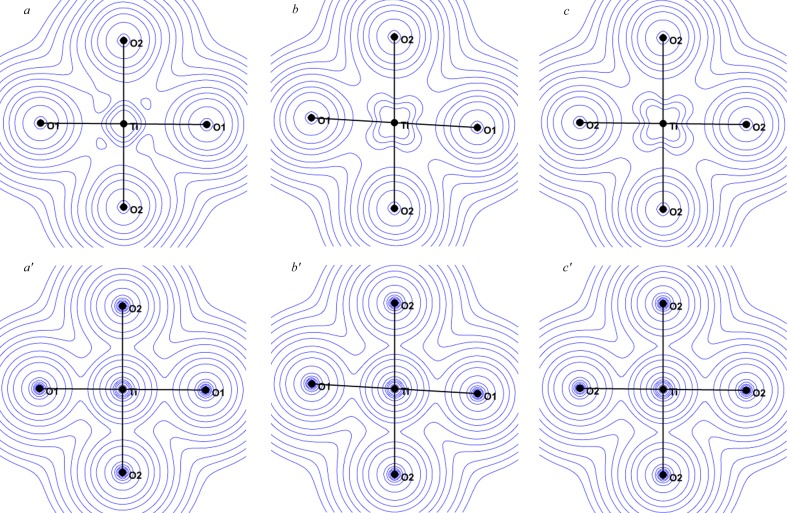
Valence density in the *xy*, *xz* and *yz* planes for spin-up (*a*, *b*, *c*) and spin-down (*a*′, *b*′, *c*′) electrons. Contour: 0.1 e Å^−3^.

The analysis of the spin resolved valence density (Fig. 11 ▸) shows that spin-down electrons evenly occupy all five 3*d* orbitals, and that all the deformation is carried out by the spin-up electrons. Such a repartition was already discussed in the end-to-end conformation of di-azido di-copper complexes (Deutsch *et al.*, 2014 ▸). This spin distribution is in partial accordance with crystal and ligand field effects that lift the degeneracy of the *t*_2g_ and *e*_g_ orbitals; the *e*_g_ orbitals oriented toward the Ti atom are 10% populated as well as the *d*_*xy*_ orbital. The spin wavefunction of the unpaired Ti electron is mainly a linear combination of *d_xz_* and *d_yz_* orbitals with a slight contribution of the other orbitals. This is consistent with the results of theoretical calculations (Mizokawa & Fujimori, 1996 ▸; Mizokawa *et al.*, 1999 ▸; Sawada *et al.*, 1997 ▸; Yan *et al.*, 2017 ▸) and with the experimental determination of the Ti wavefunction using different experimental methods such as polarized neutron scattering (Ichikawa *et al.*, 2000 ▸; Akimitsu *et al.*, 2001 ▸; Kibalin *et al.*, 2017 ▸), NMR spectroscopy (Itoh *et al.*, 2004 ▸), resonance X-ray scattering (Nakao *et al.*, 2002 ▸), XMD (Itoh *et al.*, 2004 ▸) and soft X-ray linear dichroism (Iga *et al.*, 2004 ▸). These *d* orbital fillings are fundamental information which infer to the existence of orbital ordering observed at low temperature in the ferromagnetic state of this perovskite (Suzuki *et al.*, 2007 ▸; Itoh *et al.*, 1999 ▸; Ichikawa *et al.*, 2000 ▸; Akimitsu *et al.*, 2001 ▸; Kibalin *et al.*, 2017 ▸; Yan *et al.*, 2017 ▸); however, as discussed above, this orbital ordering is not at the origin of the ferromagnetism as this it is already observed above the ferromagnetic transition at 100 K.

## 6. Conclusions   

Low-temperature high-resolution X–ray diffraction has been carried out on YTiO_3_ using the SPRING8 synchrotron radiation source. Despite the important absorption and extinction effects exhibited by the sample, a very accurate data set has been obtained. This has enabled realistic charge density modelling. At low temperature, Y and Ti atomic displacements are anharmonic. The joint refinement of X-ray and polarized neutron diffraction allowed a spin resolved experimental electron density determination. The obtained model shows that the titanium spin wavefunction can be approximated as a linear combination of *d*_*xz*_ and *d*_*yz*_ orbitals, which is in agreement with previous results (Kibalin *et al.*, 2017 ▸). The integrated spin moments of different atomic basins seem to indicate that the magnetic pathway involves both O atoms, not just one of them (Kibalin *et al.*, 2017 ▸), whereas magnetic moments calculated from *P*_val_ values do not. The experimentally modelled spin and charge density of Ti and O ions agree well with the *Crystal14* calculations. This study confirms the orbital ordering at low temperature (20 K), which is already present in the paramagnetic state above the ferromagnetic transition (100 K) (Voufack, 2018 ▸).

## Supplementary Material

Supporting figures and tables. DOI: 10.1107/S2052252519009230/gq5011sup1.pdf

## Figures and Tables

**Figure 10:**
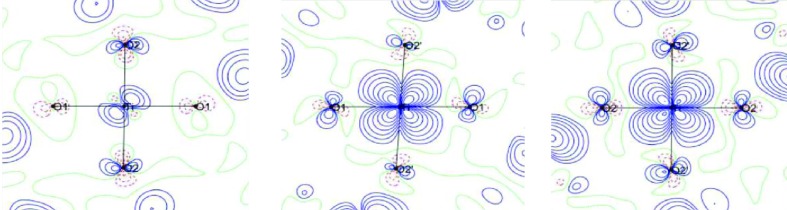
DFT spin densities in the *xy*, *xz* and *yz* planes (left to right). Contour: logarithmic 0.01 × 2^*n*^ (*n* = 1 to 12).

**Table 1:** Experimental and crystallographic data

Crystallographic data		
Chemical formula	YTiO_3_	
Space group	*Pnma*	
*a*, *b*, *c* (Å) at 20 K	5.6900 (1), 7.583 (2), 5.318 (1);	
*a*, *b*, *c* (Å) at 100 K	5.6929 (1), 7.5899 (2), 5.3241 (2)	
		
Experimental data	X-ray	Polarized neutron
μ (mm^−1^)	4.1	
Wavelength (Å)	0.3506	0.84
Absorption *T*_min_/*T*_max_	0.13/0.17	
Temperature (K)	20	5
sin θ/λ_max_ (Å^−1^)	1.668	0.5
No. of measured reflections	96917	291
No. of unique reflections†	4584	–
*R*_int_ †	3.66%	–

† PND data were not averaged

**Table 2:** Main distances and angles in YTiO_3_ *i*, *j*, *k*… are symmetry related atoms.

	Distance ( 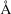 )		Angle (°)
Ti—O1	2.0164 (8)	O2—Ti—O1	89.51 (2)
Ti—O2	2.0194 (9)	O2′—Ti—O1	86.62 (2)
Ti—O2′	2.0784 (7)	O2′—Ti—O2	89.37 (4)
Y—O1	2.2343 (12)	O1—Y—O2	100.35 (2)
Y—O1*i*	2.3098 (10)	O2—Y—O2*j*	79.48 (3)
Y—O2	2.2778 (7)	O1—Y—O1*i*	88.04 (3)
Y—O2*j*	2.2778 (7)	O1*i*—Y—O2*j*	138.88 (2)
Y—O2*k*	2.5008 (9)	O2*k*—Y—O1	137.99 (2)
Y—O2*l*	2.5008 (9)	O2*k*—Y—O2	119.04 (3)
Y—O2*m*	2.6773 (10)	O2*k*—Y—O2*j*	74.98 (3)
Y—O2*n*	2.6773 (10)	Ti—O1—Ti	140.10 (2)
		Ti—O2—Ti	143.73 (2)

**Table 3:** Statistical agreement factors after the joint refinement

	XRD	PND
No. of reflections	4212	291
*R* (%)†	1.11	4.85
*R*_w_ (%)‡	1.36	3.43
(1 − *r*)%§	–	11.56
GooF¶	1.34	9.7
No. of parameters	207	27

†
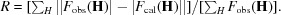

‡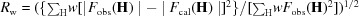. *F*_obs_ and *F*_cal_ are the observed and calculated structure factors.

§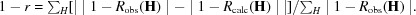 *R*_obs_ and *R*_calc_ are the experimental and calculated flipping ratios.

¶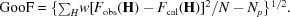 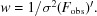

**Table 4:** Spin resolved valence populations, net charges and magnetic moments as estimated from valence populations, *Q* = *N*_val_ − *P*_val_ (in e), 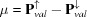 (in e) and from the AIM method *V* is the volume (Å^3^) of the atomic basin, *R* is the equivalent spherical radius *R* = [(3/4π)*V*]^1/3^. *R*_c_ is the covalent radius (Pyykko) and *R*_i_ is the ionic radius (Shannon & Prewitt, 1969 ▸).

					P_val_ monopole		Bader integration					
Atom	κ	κ’	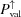	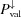	*Q*	μ	*Q*	μ	*V*	*R*	*R*_c_	*R*_i_
Y	1.03 (8)	1.49 (6)	0.76 (4)	0.78 (4)	1.46 (7)	−0.03 (7)	1.80	0.066	18.43	1.63	1.90	1.04
Ti	1.14 (2)	0.90 (3)	2.22 (4)	1.18 (4)	0.59 (6)	1.04 (6)	1.47	0.628	8.85	1.28	1.60	0.81
O1	0.964 (4)	0.88 (7)	3.34 (2)	3.32 (2)	−0.66 (3)	0.02 (3)	−1.06	0.112	10.25	1.34	0.66	1.38
O2	0.968 (2)	0.98 (7)	3.36 (1)	3.35 (1)	−0.70 (2)	0.00 (2)	−1.05	0.097	9.90	1.33	–	–

**Table 5:** Topological properties at the saddle critical points Distances are given in Å, ρ in e Å^−3^ and 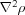 in e Å^−5^.

Bonds (*X*—*Y*)	*d*_(*X*—*Y*)_	*d*_(*X*—cp)_	*d*_(*Y*—cp)_	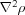	ρ(cp)
O1—Ti	2.0164 (8)	1.00	1.02	8.94	0.59
O2—Ti	2.0194 (9)	1.00	1.02	9.25	0.58
O2′—Ti	2.0784 (7)	1.02	1.07	9.10	0.47
O1—Y′	2.234 (1)	1.00	1.24	7.06	0.71
O2—Y′′	2.2778 (7)	1.00	1.27	7.02	0.62
O1—Y	2.310 (1)	1.03	1.29	4.07	0.64
O2—Y	2.5008 (9)	1.11	1.40	3.90	0.36
O2—Y′′′	2.677 (1)	1.19	1.49	2.71	0.24

**Table 6:** The *d* orbital populations obtained at 100 (charge only) and 20 K (charge and spin) including *s* contribution

Experiment	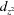	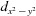	*d_xy_*	*d_xz_*	*d_yz_*
100 K	0.56, 16%	0.64, 18%	0.49, 14%	1.01, 28%	0.79, 23%
20 K charge	0.55, 16%	0.54, 16%	0.59, 17%	0.89, 27%	0.85, 25%
20 K spin	0.15, 14%	0.08, 8%	0.13, 13%	0.31, 29%	0.37, 36%
20 K spin up	0.35, 16%	0.31, 14%	0.36, 16%	0.60, 27%	0.61, 27%
20 K spin down	0.20, 17%	0.23, 19%	0.23, 19%	0.29, 24%	0.24, 20%
Pure *d* contribution	0.15, 11%	0.14, 10%	0.19, 13%	0.49, 35%	0.45, 32%
