# Supplementary material for: Spin resolved electron density study of YTiO3 in its ferromagnetic phase: signature of orbital ordering
Source: IUCrJ. 2019 Aug 2;6(Pt 5):884–94. doi: 10.1107/S2052252519009230 (PMC6760435; doi:10.1107/S2052252519009230)
Supplement: Supplementary file 1 [file m-06-00884-sup1.pdf]

IUCrJ

Volume 6 (2019)

Supporting information for article:

Spin resolved electron density study of YTiO3 in its ferromagnetic phase: signature of orbital ordering

Ariste Bolivard Voufack, Iurii Kibalin, Zeyin Yan, Nicolas Claiser, Saber Gueddida, Béatrice Gillon, Florence Porcher, Arsen Gukasov, Kunishisa Sugimoto, Claude Lecomte, Slimane Dahaoui, Jean-Michel Gillet and Mohamed Souhassou

Table S1 Statistical agreement for data averaging sorted in equal shell

Range(Å)	Sinθ/λ($Å^{-1}$)	N_{mead}	N_{unique}	N	R_1	R_2	R_W	<Q>
All	0-1.67	96986	4584	21.2	0.037	0.062	0.082	30.7
d>0.814	S<0.61	6609	240	27.5	0.031	0.070	0.031	81.2
0.814--0.646	0.61--0.77	6726	233	28.9	0.026	0.035	0.037	63.1
0.646--0.564	0.77--0.89	6688	235	28.5	0.027	0.036	0.040	55.2
0.564--0.513	0.89--0.98	6478	229	28.3	0.032	0.040	0.048	50.6
0.513--0.476	0.98--1.05	2988	190	15.7	0.056	0.055	0.080	17.5
0.476--0.448	1.05--1.12	6125	230	26.6	0.039	0.048	0.054	39.2
0.448--0.426	1.12--1.17	6060	239	25.4	0.042	0.052	0.059	33.5
0.426--0.407	1.17--1.23	5260	216	24.4	0.044	0.054	0.062	31.1
0.407--0.391	1.23--1.28	5572	240	23.2	0.049	0.060	0.067	26.9
0.391--0.378	1.28--1.32	4982	223	22.3	0.051	0.066	0.077	24.7
0.378--0.366	1.32--1.37	5017	228	22.0	0.057	0.065	0.079	22.8
0.366--0.356	1.37--1.40	4626	218	21.2	0.060	0.067	0.082	21.3
0.356--0.346	1.40--1.45	5053	246	20.5	0.066	0.079	0.089	18.8
0.346--0.338	1.45--1.48	4508	223	20.2	0.069	0.076	0.094	18.1
0.338--0.330	1.48--1.52	3974	220	18.1	0.072	0.079	0.099	16.6
0.330--0.323	1.52--1.55	3449	240	14.4	0.077	0.086	0.103	14.3
0.323--0.317	1.55--1.58	2644	211	12.5	0.086	0.098	0.111	12.6
0.317--0.311	1.58--1.61	2448	234	10.5	0.098	0.134	0.128	10.7
0.311--0.305	1.61--1.64	2349	238	9.9	0.088	0.102	0.115	10.7
0.305--0.300	1.64--1.67	1866	213	8.8	0.098	0.113	0.130	9.05

Table S2 Significant Gram-Charlier parameters for Y and Ti atoms.

C_{ijklmn}(Y)	C(σ(C))	abs(C)/σ(C)>2	C_{ijklmn}(Ti)	C(σ (C))	abs(C)/σ(C)
333	0.00016(7)	2.3	1111	-0.00045(8)	5.6
122	0.00023(7)	3.3	2222	-0.00016(2)	8.0
223	0.00016(7)	2.3	3333	0.00097(7)	13.9
2222	-0.000065(9)	7.2	1112	0.0003(1)	3.0
3333	0.00119(4)	29.8	1122	-0.0011(1)	11.0
1122	-0.00099(5)	19.8	2233	0.0004(1)	4.0
2233	0.00034(6)	5.7	2333	-0.0003(1)	3.0
12222	0.000020(6)	3.3	222222	-0.000010(2)	5.0
22333	0.00005(1)	5.0	333333	0.00009(2)	4.5
111111	0.000035(9)	3.9	111122	-0.00019(3)	6.3
333333	0.00014(1)	14.0	112222	-0.00010(2)	5.0
111122	-0.00018(1)	18.0	112233	-0.00020(5)	4.0
112222	-0.000072(7)	10.3	113333	0.00019(7)	2.7

112233	-0.00015(3)	5.0	223333	0.00021(3)	7.0
113333	0.00025(4)	6.3			
223333	0.00017(2)	8.5			

Table S3 Values of n_l and ζ_l of Slater radial function

atom	n_l	ζ_l (bohr^{-1})
Y	4,4,4,4,4	6.8
Ti	4,4,4,4,4	7.00
O	2,2,2,3,4	4.46

Table S4 Atomic fractional coordinates and atomic displacement parameters (Uij*10^5)

	x	y	z	U11	U22	U33	U12	U13	U23
Y	0.073950(7)	0.25	-0.021949(9)	192(2)	189(1)	237(1)	0	-6.9(7)	0
Ti	0.5	0.5	0	214(2)	146(2)	222(2)	3(2)	-3(1)	3(1)
O1	0.45749(7)	0.25	0.12105(8)	394(9)	266(8)	38(1)	0	63(7)	0
O2	0.30942(5)	0.44209(4)	-0.30944(6)	371(7)	412(7)	374(7)	62(5)	-62(5)	-43(5)

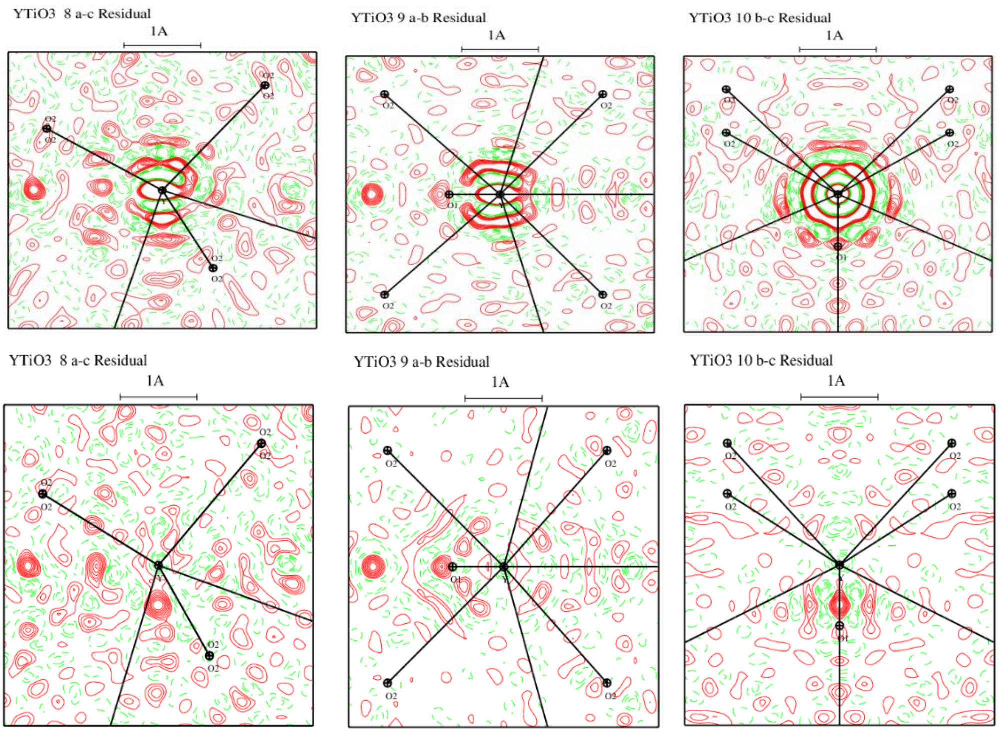

Figure S1 Residual density at high angle (1.4 < sin(θ)/λ < 1.67 A^{-1}), in ab,bc and ac plane containing Y atom. contour 0.1 e /A^3. Upper: Harmonic model for Y and Ti. Down: Anhamonic model

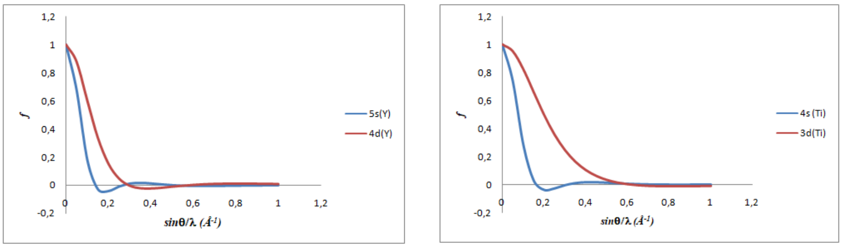

Figure S2 Normalized 5s and 4d valence scattering factor of Y (left) and Normalized 4s and 3d valense scattering factor of Ti(right).

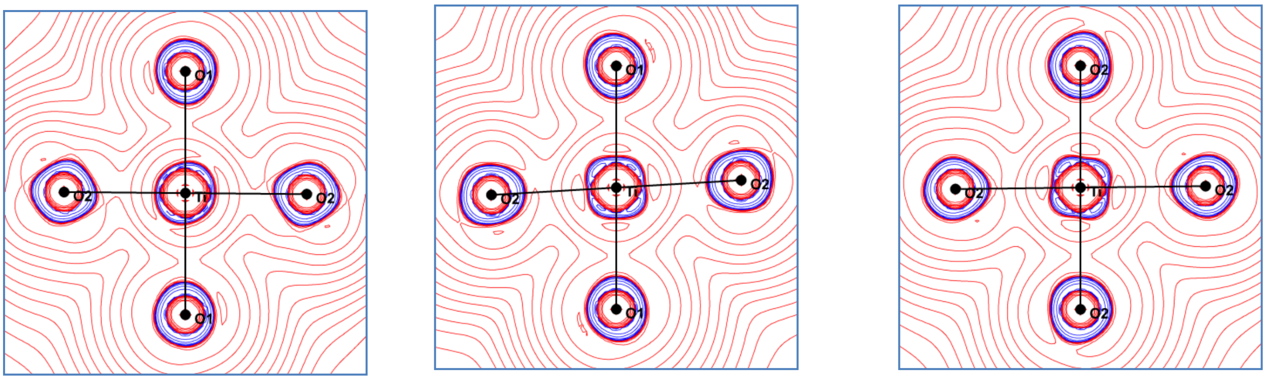

Figure S3 Negative Laplacian of the density in logarithmic scale in xy, xz and yz planes contour 10 e/Å^5

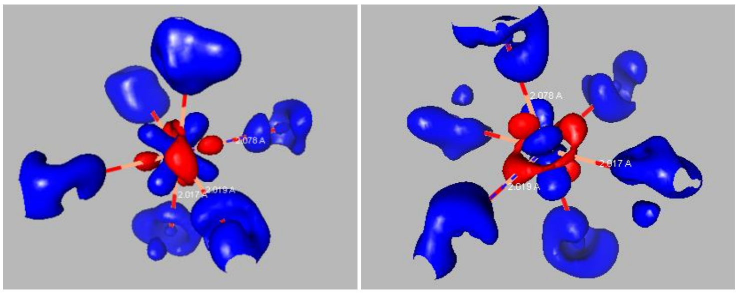

Figure S4 Static deformation density around Ti atom. Isosurface 0.05 e /A^3
